# Strain Effect on Dielectricity of Elastic Thermoplastic Polyurethanes

**DOI:** 10.3390/polym16111465

**Published:** 2024-05-22

**Authors:** Yubo Wang, Huali Yang, Yali Xie, Xilai Bao, Lili Pan, Dan Zhao, Jinxia Chen, Mengting Zou, Tian Tian, Runwei Li

**Affiliations:** 1 School of Materials Science and Engineering, Shanghai Institute of Technology, Shanghai 201400, China; 2 CAS Key Laboratory of Magnetic Materials and Devices, Ningbo Institute of Materials Technology and Engineering, Chinese Academy of Sciences, Ningbo 315201, China; xieyl@nimte.ac.cn (Y.X.); zhaodan@nimte.ac.cn (D.Z.); runweili@nimte.ac.cn (R.L.); 3 Zhejiang Province Key Laboratory of Magnetic Materials and Application Technology, Ningbo Institute of Materials Technology and Engineering, Chinese Academy of Sciences, Ningbo 315201, China

**Keywords:** dielectric constant, dielectric elastomer, polyurethane, strain, hard domains

## Abstract

Dielectric elastomers, such as thermoplastic polyurethanes (TPUs), are widely used as the dielectric layer, encapsulation layer, and substrate of flexible and stretchable devices. To construct capacitors and actuators that work stably upon deformation, it has become urgent to investigate the evolution of dielectricity under stress and strain. However, the lack of effective methods for estimating the dielectric constant of elastomers under strain poses a big challenge. This study reports a device for the in situ measurement of the dielectric constant of TPU under strain. It is found that upon stretching TPU to a strain of 400%, its dielectric constant decreases from 8.02 ± 0.01 to 2.88 ± 0.25 (at 1 MHz). In addition, combined Fourier-transform infrared spectroscopy, the X-ray scattering technique, and atomic force microscopy were utilized to characterize the evolution of the microstructure under strain. The investigation under tensile strain reveals a decreased density and average size of polarized hard domains, along with a tendency of the molecular chains to align in parallel with the tensile stress. The evolution of the microstructures results in a reduction in the measured dielectric constant in TPU.

## 1. Introduction

In recent years, the rapid development of the metaverse, human–computer interaction, intelligent medical technology [1], and other industries has propelled the advancement of flexible electronic devices and actuators. As the foundation of flexible devices, various functional materials that are flexible and even stretchable are emerging rapidly, among which dielectric elastomers are widely investigated due to their essential role in constructing flexible sensors, circuits [2], transistors [3], and artificial muscle. For example, thermoplastic polyurethane (TPU) is considered a crucial component of flexible capacitors [4], which plays a vital role in pressure sensing [5] and displacement measurement. Additionally, capacitors enable diverse functions, such as tuning, by-passing, coupling, and filtering, when employed within logic circuits [6]. Moreover, artificial muscle made of dielectric elastomers can deform when an electric field is applied, which could be used for prosthetics [7], surgical robots, and wearable devices [5], as well as soft robots [8] capable of locomotion and manipulation in natural environments or inside the human body. 

In the above scenario, dielectric elastomers typically need to operate while in deformation. Characterizing the dielectric properties during the strain process is not only beneficial for providing an important reference for improving the tensile stability of flexible capacitors [9] but also helps to improve the accuracy of modeling electrical parameters such as energy density and power density [10,11] for actuators under strain conditions. Due to the significant impact of different dielectric constants under strain on the electromechanical coupling of devices, studying the regulation of dielectric constants of dielectric elastomers under stress is crucial.

Previous studies have shown that the dielectric constant can indeed be affected by an external strain [12,13,14,15,16,17,18]. For example, Vu-Cong et al. [19] and Wissler et al. [20] experimentally found that the dielectric constant of VHB4910 decreased at stretching deformation. However, the traditional approach for determining the dielectric constant upon stretching is ex situ and clumsy. In these approaches, samples with a predetermined area were stretched by clamping them in a frame, following which the electrodes were prepared and the capacitance was measured to determine the dielectric constant [21,22,23]. Based on the above methods, the sample thickness measurement is not accurate, and the tensile strain is difficult to measure accurately [18]. Therefore, it is necessary to construct an experimental setup that enables the in situ and accurate determination of the dielectric constant upon stretching. Regarding the theoretical aspects, many models have been constructed to predict the dielectric constant of elastomers under strain [24,25,26], where the polarization degree [27] and orientation distribution of dipoles [28] in elastic materials were considered to process as factors. However, experimental evidence of the in situ characterization of the structure evolution of an elastomer upon strain is still lacking. Herein, we proposed and designed a device capable of measuring the permittivity of TPU in situ under different strain states. We quantitatively determined its dielectric constant at different strains up to 400%. Moreover, by employing micro-polarization theory along with techniques, such as wide-angle X-ray scattering (WAXS), small-angle X-ray scattering (SAXS), Fourier-transform infrared spectroscopy (FTIR), and atomic force microscopy (AFM) analysis, a link between the evolution of the dielectric constant and the microstructures under external strain was found. TPU is mainly composed of soft segments (polyester, polyether polyol) and hard segments (diisocyanate) [29], which are formed through a chain extender polycondensation reaction. Our results indicate that the density and average size of polarized hard domains decrease upon stretching, and the molecular chains tends to align in parallel with the tensile stress. As the polarity of TPU is predominantly provided by the hard segment phase [30,31,32], such evolution of the microstructures mainly accounts for the decrease in the dielectric constant in TPU.

## 2. Materials and Methods

### 2.1. Dielectric Elastomers

The TPU (product name: TPU-85A) used in this study was provided by Jiaxin Plastic Material Company (Dongguan, China). This TPU is mainly composed of an amorphous soft segment formed of a polyether polyol based on propane diol (WANOL^®^ C2010D) and a hard segment formed of 4,4′-diphenylmethane diisocyanate (MDI, WAN-NATE^®^MDI-100). The infrared spectrum, mechanical properties, and differential scanning calorimetry (DSC) curve of the purchased TPU are presented in Figures S1–S3 (Supplementary Information).

### 2.2. Experimental Setup

**Figure 1:**
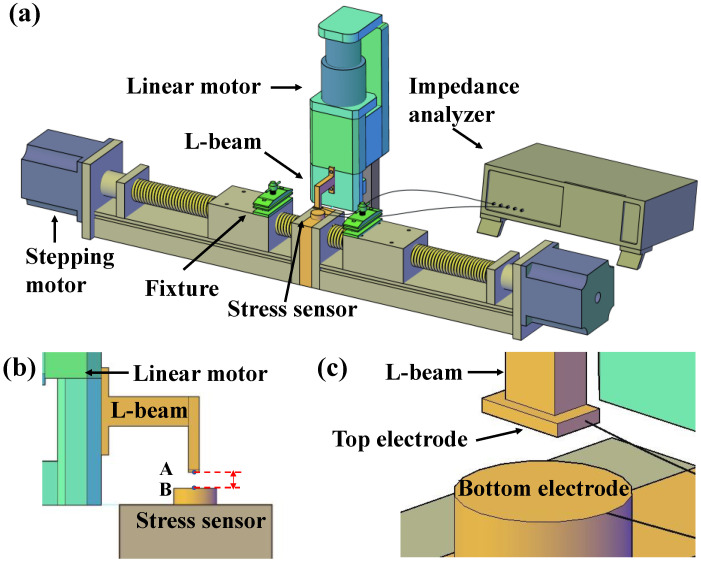
Experimental setup and the effect of strain on dielectric constant. (**a**) Diagram of a device for measuring the dielectric constant of an elastomer under tension strain. (**b**) Side view of the thickness measurement part. (**c**) Enlarged view of the electrodes.

The device for measuring the dielectric constant of elastomers under strain is mainly based on the principle of parallel-plate capacitor proposed by Hermann von Helmholtz. The essence of this principle is to calculate the dielectric constant value of the material by measuring the capacitance and shape of the material:

ε_r_ = Cd/(ε_0_ S) (1)

where C is the capacitance, ε_0_ is the vacuum dielectric constant, ε_r_ is the relative dielectric constant, S is the area opposite the electrode plate, and d is the thickness of the dielectric layer. As is shown in Figure 1a, the device has three main components: the mechanical stretching system, the thickness measurement system, and the capacitance measurement system. The mechanical stretching system consists of two stepper motors (model: 57HBP56AL4-TFA8B, supplier: Beijing Era Super Electric Technology Co., Ltd., Beijing, China) placed on the same axis with the same speed but opposite direction of motion. Each stepper motor is equipped with a DC programmable intelligent servo driver, and the two motors are controlled by a controller to achieve synchronous movement. The dielectric elastomer is fixed on each end of the stepper motor by a clamp, and transverse stretching is applied onto the sample in a controllable manner. The original length a (fixture to fixture) of the sample to be stretched is a = 33 mm. The total effective tensile length of the system is 424 mm, and the tensile accuracy is 4 ± 0.5 mm. To reduce the residual stress in the sample as much as possible, the samples are slowly stretched at a speed of 0.1 mm/s. In contrast to previous methods for measuring dielectric constants, our setup enables the measurement of elastomer dielectric constants under arbitrary tensile strains. To set the strain of the specimens, we set the elongation (∆a) of the specimens by controlling the turning rounds of the stepper motor; then, the strain could be estimated by ε = (∆a/a) × 100%.

Accurate thickness measurement is another crucial issue in determining the dielectric constant. In order to accurately measure the thickness of an elastomer, the force is usually applied on the elastomer surface. In light of this situation, we integrated the pressure and displacement sensors into the upper and lower electrode plates, so as to measure the changes in elastomer thickness more accurately. The thickness measurement system consists of a pressure sensor (model: F11, supplier: Changzhou Fabitai Intelligent Technology Co., Ltd., Changzhou, China), voice coil displacement sensor (model: LMHC-13-S20, supplier: Shenzhen Daqian Technology Co., Ltd., Shenzhen, China), control software program (V1.6), and an L-shaped cantilever beam. The pressure sensor has a comprehensive accuracy of 0.5 F.S. and a repeatability error of less than 0.2% in a force range of up to 10 N. The voice coil displacement sensor has an accuracy of 0.001 mm, with a selected motion rate of 0.1 mm/s. The working process involves initially zeroing the distance between the upper and lower plates. As the upper plate moves downward and gradually increases the contact force up to a pre-set value of 0.1 N, it immediately stops moving. At this point, the voice coil displacement sensor allows us to estimate the change in sample thickness upon stretching (Figure 1b). 

In addition, the measurement of capacitance is also important for the measurement of permittivity, and the shape and material of the electrode plate directly affect the measurement of capacitance. As a result of the fact that flexible electrodes deform upon stretching, rigid electrodes were utilized. In addition, the upper electrode was composed of a rectangular brass of 5.5 mm × 2.8 mm × 1 mm, and the lower plate was composed of a circular brass sheet with a diameter of 20 mm attached to the surface of the pressure sensor (Figure 1c). In this way, the area of the electrode is determined by the upper electrode plate. The capacitance value was recorded by using a Hioki IM3570 impedance analyzer (HIOKI, Yokohama, Japan) with 2-point to 4-point measurement type. A photograph of the setup is shown in Figure S4 (Supporting Information). Generally, the measured capacitance usually contains contributions from the sample and other factors, such as air, stray capacitance, and conductors between the plates. In order to validate the reliability of the experimental setup, the setup was calibrated by using standard sample prior measurements, as described in Note S1 (Supporting Information).

### 2.3. Characterization

**Figure 2:**
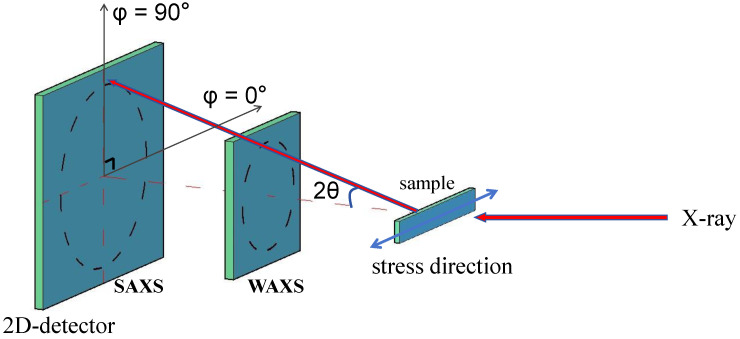
Schematic diagram showing the experimental configuration of the wide-angle X-ray scattering (WAXS) and small-angle X-ray scattering (SAXS).

SAXS and WAXS experiments were conducted at room temperature using a Xeuss 3.0 UHR system (Xenocs, Grenoble, France), which emitted Cu-Kα radiation with a wavelength of 1.54 Å under a voltage of 50 kV and a current of 0.6 mA. The experiments were carried out at ambient temperature using an Eiger 2R 500K detector (Bruck, Billerica, MA, USA). The distances from the samples to the detector for the WAXS and SAXS tests were 60 mm and 1200 mm, respectively. The exposure times were set to 600 s for SAXS and 180 s for WAXS. The tensile tester used was a Linkam MFS350 (Linkam Scientific Instruments, Redhill, UK). During measurement, the sample was first stretched to a predetermined strain and then exposed for a period to collect images. A schematic representation of the sample detector orientation is included in Figure 2 to illustrate the relative positions of the stretching direction and the detector clearly.

The infrared spectroscopy (ATR-FTIR) testing was performed using the Agilent Cary 660 FTIR instrument from Agilent Technologies (Santa Clara, CA, USA), equipped with an ATR unit (MIRacle, PIKE Technologies, Inc., Madison, WI, USA) and a DTGS detector. All spectra were collected with 32 scans, a resolution of 4 cm^−1^, and a scanning range from 340 to 4000 cm^−1^.

The atomic force microscopy (AFM) was characterized by using tapping mode on a Dimension ICON AFM (Bruker, Billerica, MA, USA). The AFM tip (Model: RTESP-150) has a spring constant of 5 N/m and a resonance frequency of 150 kHz.

## 3. Results

### 3.1. Variation in the Dielectric Constant of TPU under Tensile Strain

**Figure 3:**
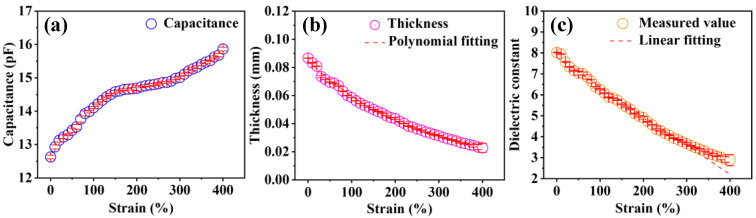
Variation in the TPU capacitance (**a**), thickness (**b**), and dielectric constant (**c**) with strain.

After calibration, the device was used to measure the capacitance variation in TPU with strain and the thickness variation in TPU with strain, and the variation in the relative permittivity with strain was calculated. Based on the experimental setup diagram, we supplemented the dielectric spectrum and the tangent of the dielectric loss of TPU at different strain states, ranging from 1 kHz to 5 MHz (Figure S7). It can be seen that the dielectric constant continuously decreases with increasing strain, while the tangent of the dielectric loss gradually increases. At the same strain level, the dielectric constant also shows a slow decrease with increasing frequency. In specifically, under test conditions of temperature at 20 °C, frequency at 1 MHz, and voltage at 1 V, it was observed that the TPU capacitance gradually increased with increasing strain (Figure 3a). When the strain was 0%, the initial capacitance obtained through the calibration method was 12.4 pF, with an error bar of 0.05 pF. Meanwhile, the variation in TPU thickness during the stretching process was measured, as shown in Figure 3b. The initial thickness value was 0.0866 mm and gradually decreased to 0.0227 mm when the strain reached 400%. At a strain of 380%, the error bar of the thickness value suddenly increased, as the film thickness approached the limit of the sensor (0.001 mm). Therefore, a strain measurement range of 0% to 400% was chosen.

Finally, the variation in relative permittivity with strain was calculated using the parallel-plate capacitor principle, as shown in Figure 3c. The results indicated that with increasing strain, the relative permittivity gradually decreased from 8.02 ± 0.01 (ε = 0%) to 2.88 ± 0.25 (ε = 400%). It was found that the relative permittivity of TPU decreased gradually with increasing strain. The linear fitting of the curve yielded a goodness-of-fit value of R^2^ = 98.3%. 

### 3.2. Orientation Behavior of TPU during Tensile Strain

**Figure 4:**
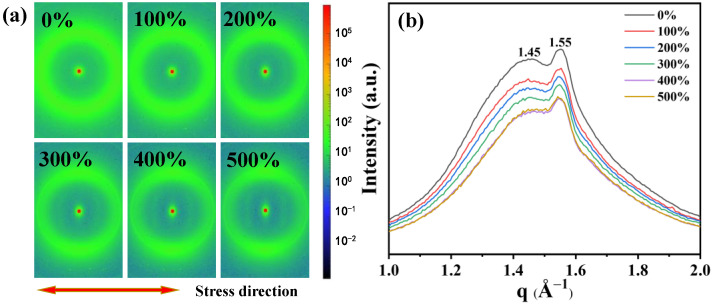
Effect of strain on TPU molecular chain. (**a**) WAXS of TPU at various strains. (**b**) One−dimensional WAXS curves azimuthally integrated with TPU under different tensile strains.

The dielectric constant is a macroscopic manifestation of the polarization ability of a material. Orientational polarization is a common polarization mode in dielectric materials [33], and strain can induce orientational changes in TPU molecular chains [34]. The polarization ability and dielectric constant can be influenced by the formation of crystallization or orientation in the molecular chains under certain conditions [9]. To gain a deeper understanding of the orientation behavior of TPU during stretching, we used in situ WAXS to test the microstructure of TPU in different stretching states. As shown in Figure 4a, when not stretched, the scattering ring of TPU is a concentric circular Debye ring, indicating an isotropic and disordered distribution of the molecular chains. As the strain reaches 100%, the scattering ring of TPU gradually becomes elliptical, indicating that there is orientation occurring between the molecular chains. According to the regions where scattering rings appear, it can be inferred that the molecular chains tend to orientate along the stretching direction. As the strain increases to 200%, diffuse spots appear in the scattering ring, and at 500%, more distinct diffraction spots concentrated in the direction perpendicular to the stretching direction can be observed, further indicating the occurrence of orientation behavior in the molecular chains of TPU after stretching.

Figure 4b displays the one-dimensional WAXS curve, which shows the distribution of weak crystal scattering peaks under different stretching states. When the strain is 0%, two scattering peaks appear at q = 1.45 Å^−1^ and q = 1.55 Å^−1^, corresponding to interchain distances (d) of 4.3 Å and 4.0 Å, respectively. During the gradual increase in strain, we found that the positions of the two peaks did not show significant changes, indicating that there was no crystal phase transformation in TPU after stretching [35]. 

### 3.3. Changes in Hydrogen Bonding under Tensile Strain

The overall polarization is mainly determined by the hard domains in the TPU structure [36]. By observing changes in hydrogen bonds under strain, the variations in the structure of hard domains can be reflected [29,34]. There are three types of hydrogen bonds in the molecular conformation of TPU. Firstly, the hydrogen bonds between the hard segments in the hard domain structure are typically reflected by the carbonyl peaks in the Fourier-transform infrared spectroscopy (FTIR) spectra, expressed as “H-bonded C=O”. Secondly, the hydrogen bonds formed between the interactions of soft and hard domains are referred to as the hydrogen bonds between the hard and soft segments, which are usually reflected by the N-H peaks in the spectra, expressed as “H-bonded N-H”. Thirdly, there are unconstrained free hydrogen bonds [35]. Figure S8 (Supporting Information) shows the FTIR spectra of TPU under different states as the tensile strain increases from 0% to 400%. At ε = 0%, C-O-C asymmetric and symmetric stretching peaks can be clearly observed at 1220 cm^−1^, indicating the presence of polyether-type polyurethane. For polyether-type polyurethanes, a large number of hydrogen bonds exist near the amino acid groups. The N-H bonds in the main hard segment structure, such as the urethane groups, act as bridges to form hydrogen bonds between the hard and soft segments, combined with the C=O bonds in the hard segments to form (C=O---N-H) hydrogen bonds and combined with the C-O bonds in the soft segments to form (C-O---N-H) hydrogen bonds. However, the stretching state often increases steric hindrance in the molecular chains, affecting the formation of these two types of hydrogen bonds. Therefore, we used the attenuated total reflectance (ATR) mode in FTIR to fit and analyze the C=O peak and investigate the microphase separation behavior.

**Figure 5:**
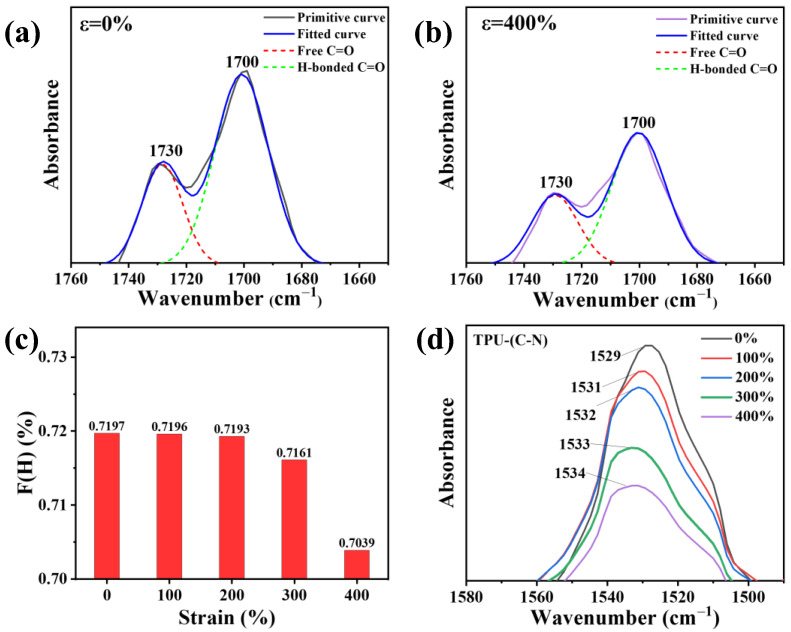
The FTIR measurements of TPU in different strain states. (**a**,**b**) FTIR spectra at strain of 0% (**a**) and 400% (**b**), respectively. (**c**) Evolution of the coefficient F(H) with strain. (**d**) FTIR spectra corresponding to C-N bond under various tensile strains.

As shown in Figure 5a,b, the peak at 1700 cm^−1^ is attributed to the hydrogen-bonded carbonyl stretching vibration, while the peak at 1730 cm^−1^ is attributed to the free carbonyl stretching vibration (more data are displayed in Figure S9 (Supporting Information)). The areas (A) of these two peaks were obtained by fitting and calculating using Gaussian functions, and the fitted curves correspond well to the original curves. Here, the coefficient F(H) representing the degree of hydrogen bond formation was obtained using the formula F(H) = A_1700_/(A_1730_ + A_1700_) × 100%. A_1700_ is the spectral peak area at 1700 cm^−1^, and A_1730_ is the spectral peak area at 1730 cm^−1^ [29,37]. As the strain increases from 0% to 400%, the values of F(H) gradually decrease (Figure 5c), which indicates that as the strain increases, the ability of microphase separation increases, leading to a gradual disruption of hydrogen bonds in the hard segments. This forces the highly polar hard domain structures to disperse within the less polar soft domain structures [38].

Simultaneously, the peak at 1529 cm^−1^ is attributed to the hydrogen-bonded C-N stretching vibration in the hard domains [39], which is also an important indicator for estimating the proportion of hard domains. As can be seen from Figure 5d, the intensity of the C-N peak decreases with increasing tensile strain. The peak area decreases from 4.336 (ε = 0%) to 1.970 (ε = 400%), indicating a gradual decrease in the number of hydrogen bonds connected to C-N as the strain increases. Interestingly, in addition to the decrease in peak intensity, a peak shift towards higher wavenumbers also occurs, suggesting that after hydrogen bonds break in the hard segments, they transform into interactions between soft domains. As a result, the frequency of C-N stretching vibrations in the hard domains increases.

### 3.4. Variation in the Molecular Chain Long Period during Stretching

**Figure 6:**
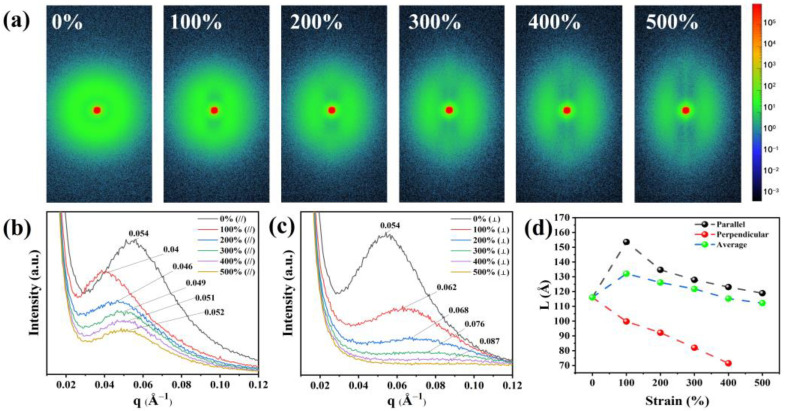
(**a**) The SAXS of TPU in different stretching states. (**b**) SAXS integrated parallel to the tensile direction. (**c**) SAXS integrated perpendicular to the tensile direction. (**d**) Estimated change in long period (L) under different strain.

To analyze the reason for the smaller number of hydrogen bonds between highly polar hard domains in TPU under strain, we investigated the evolution of the distances between hard domains and that between hard and soft domains upon different strain states in TPU molecules. The variation in the molecular chain long period (L) in TPU was studied using the SAXS technique (Xenocs, Grenoble, France). Figure 6a shows the 2D-SAXS patterns of TPU in various strain states. To obtain clear and accurate images, we stretched the sample to the specified strain value and then captured the image. At zero strain, a uniform circular scattering ring is observed around the central red spot, indicating an isotropic arrangement between the hard segments and the soft segments. As the strain increases to 100%, the scattering pattern changes from circular to elliptical, indicating the occurrence of orientation behavior in the molecular chains and the appearance of striped scattering regions perpendicular to the stretching direction. In addition, a diffuse scattering pattern appears parallel to the stretching direction. As the strain increases, the width and intensity of the diffuse scattering increase, indicating a greater degree of dispersion, while the intensity of the striped scattering region becomes stronger, indicating an increase in the number of molecular chain segments oriented in the stretching direction.

To quantitatively determine the specific changes in the hard and soft domains in the microphase separation structure, we integrated the 2D-SAXS patterns parallel and perpendicular to the stretching direction to obtain the 1D-SAXS intensity distribution in Figure 6b,c, respectively. The molecular chain long period (L) was calculated using the formula L = 2π/q_max_ (where q_max_ is the q value at the maximum intensity of the scattering peak). Figure 6b depicts the results along the stretching direction, from which the coherent scattering peak shifts to lower q values. L along the stretching direction increases from 11.6 nm (ε = 0%) to 15.4 nm (ε = 100%) as the strain increases (Figure 6d). As the strain further increases from 100% to 500%, the coherent scattering peak shifts back to higher q values, and L gradually decreases to 11.9 nm (ε = 500%), indicating that the distance between hard domains in TPU initially increases and then decreases in the stretching direction. At the same time, the intensity of the coherent scattering peak gradually decreases, indicating a reduction in the density of the hard domains. On the other hand, the long period perpendicular to the stretching direction generally decreases from 11.6 nm (ε = 0%) to 7.1 nm (ε = 500%) (Figure 6d), and the scattering intensity decreases significantly. According to the analysis of the hydrogen bonding strength in the hard segment after 100% strain in FTIR, it can be inferred that this may be due to the elastic deformation of the molecules at the beginning of stretching, where hydrogen bonds are not broken and the distance between hard domains increases horizontally. However, as the hydrogen bonds are disrupted, the hard domains evolve. From the weakening of the coherent scattering peak intensity, it can be speculated that there is an increment in the degree of microphase separation in TPU. All in all, the number of hard segments in the perpendicular direction significantly decreases, transforming into hard domains along the stretching direction. This change can also be seen from the change in azimuth angle, as shown in Figure S10 (Supporting Information). At ε = 0%, the hard domains in TPU exhibit isotropic characteristics. For ε = 100%, by defining the stretching direction as 0°, the hard domains are more likely to be oriented in the directions of 45°, 132°, 227°, and 314°. For ε larger than 100%, more and more hard domains are concentrated in the parallel stretching direction, while a small number of hard domains are perpendicular to the stretching direction. For example, they peaked at 89° and 270° at ε = 500%. This is consistent with the results of the 2D-WAXS test, which show that the molecular chains in TPU exhibit a preferential orientation along the stretching direction.

### 3.5. Characterization of TPU Microphase Separation

**Figure 7:**
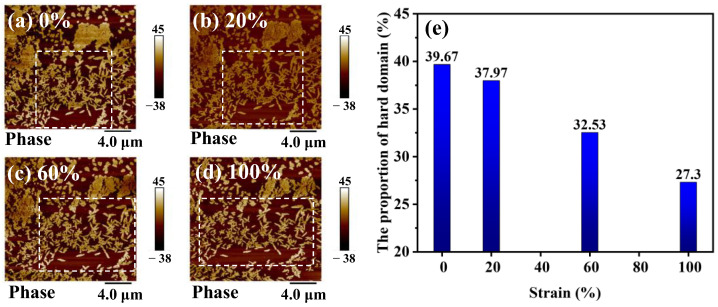
AFM images of TPU under different tensile strains. (**a**–**d**) Phase images at strain of 0% (**a**), 20% (**b**), 60% (**c**), 100% (**d**). (**e**) The proportion of hard domains under different strains.

The structure of the hard segment strongly affected the dielectric behavior [40]. To characterize the microscopic phase separation upon strain in the TPU elastomers, the AFM technique was employed to map the phase images of TPU in various strain states. We used Photoshop image processing software (Version number: 25.7) to determine the proportion of hard domains. In the image, regions with higher brightness represent hard domains, while regions with lower brightness represent soft domains. The proportion of hard domains (S) is defined as the ratio of the bright areas (S_1_) to the total area of the designated region (S_0_): $S=\frac{S_{1}}{S_{0}}\times 100\%$. As can be seen from Figure 7, the elastomer is composed of regions characterized by distinct bright and dark phase contrast. The bright regions show clear and regular edges and could, therefore, be regarded as hard domains. The percentage of hard domains in the images was calculated using image analysis software (Version number: 25.7), and the number of hard domains in the stretching process was obtained quantitatively. In the unstretched initial state, the hard domain phases are typically of a few hundred nanometers in size and show leaflet-like patterns, with more agglomerates intertwined and fewer independently present in the soft domains. As the tensile strain increases, it is vividly observed that the agglomerated hard domains are gradually dispersed into smaller independent fragments, and the lateral distances between the original independent hard domains gradually increase (Figure 7a–d). We estimated the proportion of hard domains in the same region (marked by dashed box) and found that it decreases from 39.67% to 27.30% as the strain increases from zero to 100% (Figure 7e). Our observation also provides strong support for the analytical results of SAXS.

**Figure 8:**
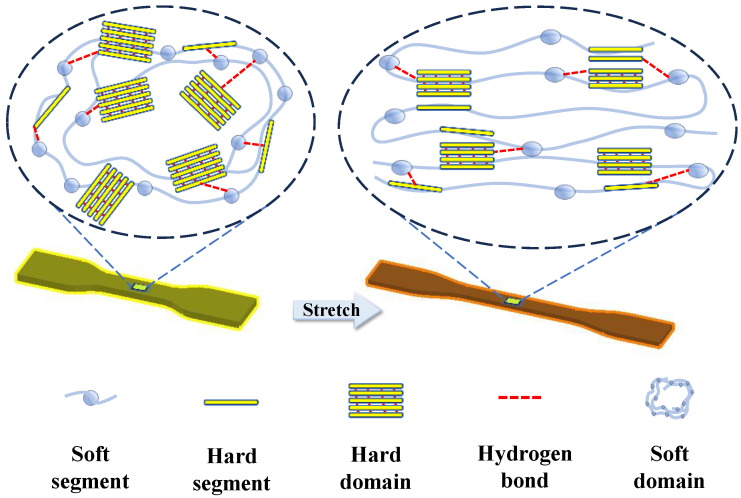
Schematic diagram showing the effect of stretching on the TPU molecular chains.

Figure 8 is a schematic diagram, showing how the microstructure of TPU evolves upon the application of external strain. Although no evidence of strain-induced phase transition was observed in TPU, there was a clear sign of the re-orientation of the molecular chains aligning along the stretching direction. Furthermore, the hard domains are gradually disrupted and enveloped by soft domains, giving rise to a simultaneous decrease in the density of the hard domains. As the hard domains contribute significantly to the dielectric constant, such convolution of hard domains shall contribute to a decrease in the observed dielectric constant of TPU.

## 4. Conclusions

In summary, we designed a device based on the parallel-plate capacitor principle for the in situ measurement of the dielectric constant of elastomers under controllable strain conditions. We measured the evolution of the dielectric constant of TPU in different strain states and found that the dielectric constant gradually decreased from 8.02 ± 0.01 (ε = 0%) to 2.88 ± 0.25 (ε = 400%) (at 1 MHz). To explain this interesting phenomenon, we characterized the effects of strain on TPU at the nanoscale by using microscopic characterization methods to observe the molecular chain spacing, hydrogen bonding status, and molecular chain long period. These characteristics indicate that during the process of increasing the strain, there is a decrease in the volume density and average size of the hard domains, and the molecular chains are aligned along the stretching direction. Such evolution of the microstructures shall contribute to a reduction in the measured dielectric constant in TPU.

## Data Availability

The data that support the findings of this study are available upon request from the corresponding author. The data are not publicly available due to privacy and legal reasons.

## Supplementary Materials

The following supporting information can be downloaded at: https://www.mdpi.com/article/10.3390/polym16111465/s1, Figure S1: The infrared spectrum of the TPU; Figure S2: The strain-stress curve of the TPU; Figure S3: Differential scanning calorimetry (DSC) curve of the TPU; Figure S4: Photograph of the experimental device for testing dielectric constant under tensile strain; Figure S5: Diagram of the open-circuit calibration and load offset calibration; Figure S6: Fluorine elastomer standard sample capacitance test; Figure S7: The frequency dependent dielectric constant (a) and loss angle tangent (b) under different strain; Figure S8: FTIR of TPU under different stretching strain (ε); Figure S9: FTIR spectra of TPU at strain of 100% (a), 200% (b), and 300% (c), respectively; Figure S10: Azimuth angle dependence of the 1D-SAXS intensity of TPU under different strains; Table S1: Displacement of scattering peaks (q = 1.45 Å^−1^ and q = 1.55 Å^−1^) under different tensile states.
