# Supplementary material for: Strain Effect on Dielectricity of Elastic Thermoplastic Polyurethanes
Source: Polymers (Basel). 2024 May 22;16(11):1465. doi: 10.3390/polym16111465 (PMC11174381; doi:10.3390/polym16111465)
Supplement: Supplementary file 1 [file polymers-16-01465-s001.zip › polymers-2966320-supplementary.pdf]

Supplementary Information

Strain Effect on Dielectricity of Elastic Thermoplastic Polyurethanes

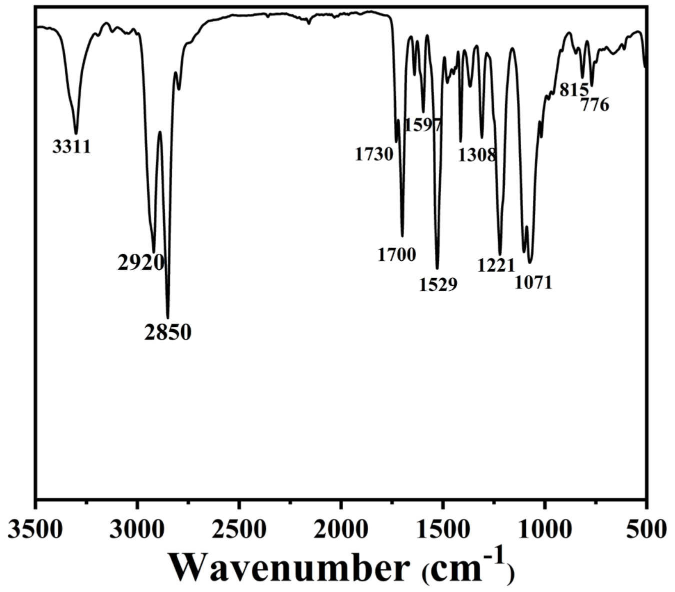

Figure S1. The infrared spectrum of the TPU.

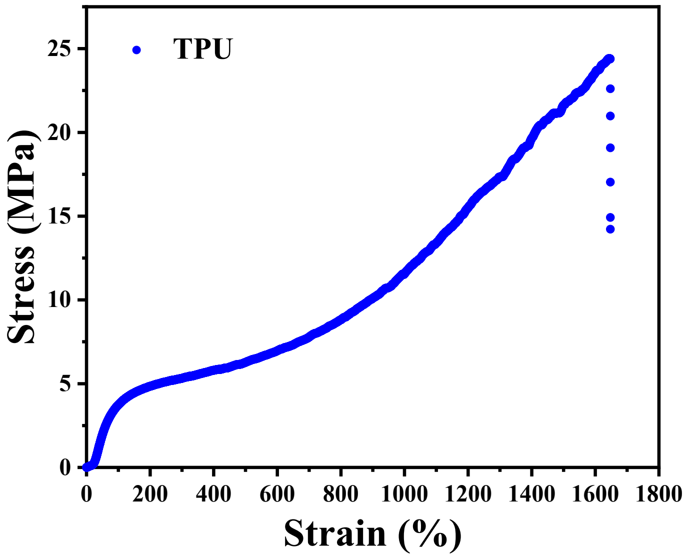

Figure S2. The strain－stress curve of the TPU.

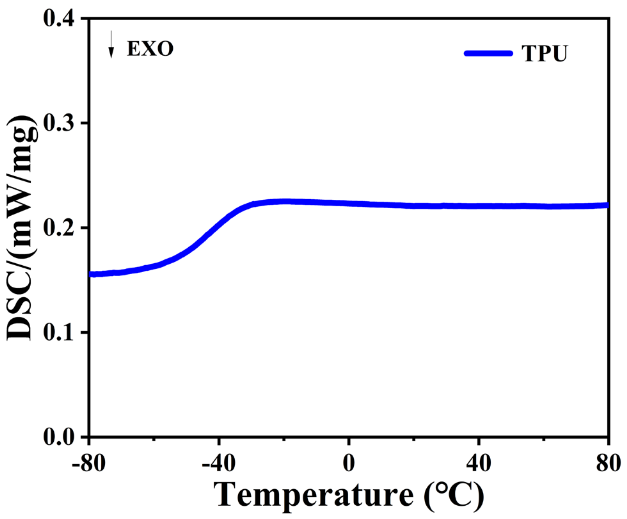

Figure S3. Differential scanning calorimetry (DSC) curve of the TPU.

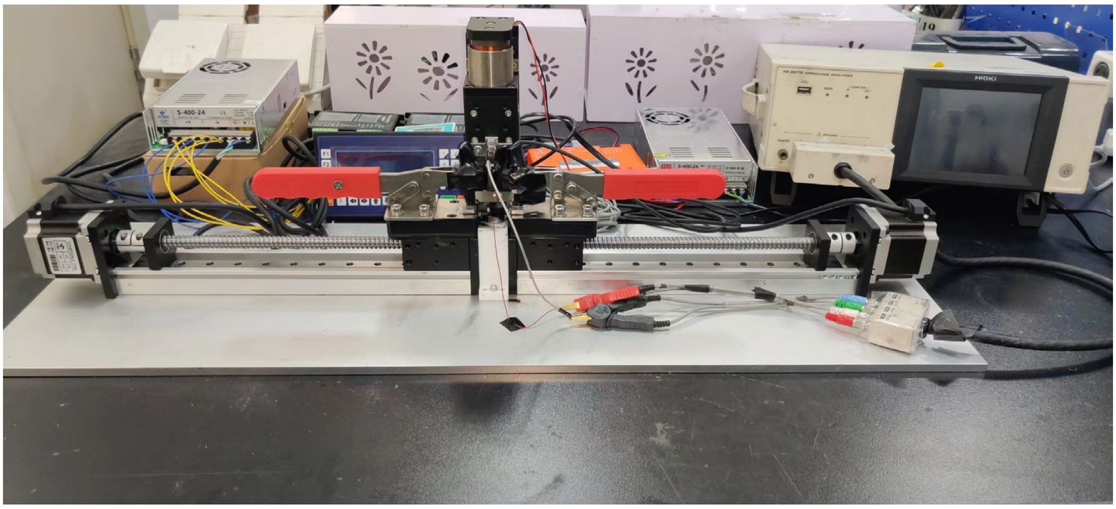

Figure S4. Photograph of the experimental device for testing dielectric constant under tensile strain.

Note S1: Instrument calibration process

1. Parameter Settings

Before calibration, we determined the test conditions for the LCR impedance analyzer. The test temperature was 20 °C, the test voltage was 1 V, and the scanning speed was "slow scan". We set the measuring mode as complex capacitance.

2.1 Instrument calibration

Short and open calibration was performed to compensate for the residual capacitance of the test fixture and cables.

(a) Short-circuit calibration

After making contact between the upper and lower electrodes, we selected the short-circuit calibration button on the impedance analyzer. The system automatically performs the short-circuit calibration.

(b) Open-circuit calibration

The area of the electrode plates in our setup is noticeable. The open-circuit capacitance (C_{open}) is mainly composed of two parts: the capacitor between the electrode plates (C_{plate}) and the capacitor between the cables (C_{cable}). When the sample is loaded, the capacitor of the sample (C_x) can be expressed as:

$$C_x = C_M - C_{cable}, \qquad (2)$$

where C_M is the measured capacitance. In order to minimize the contribution of C_{plate} in the open-circuit calibration, we lift the upper plate until it is far away from the lower plate (the distance between them is 50 mm), measuring the open capacitance value C_{open} = 1.37 pF. Notice that as the distance between electrode plates is much larger than the thickness of the elastomer sample (50 mm >> 0.0866 mm), the above open-circuit calibration is not complete. Therefore, we further use a standard sample to calibrate the system.

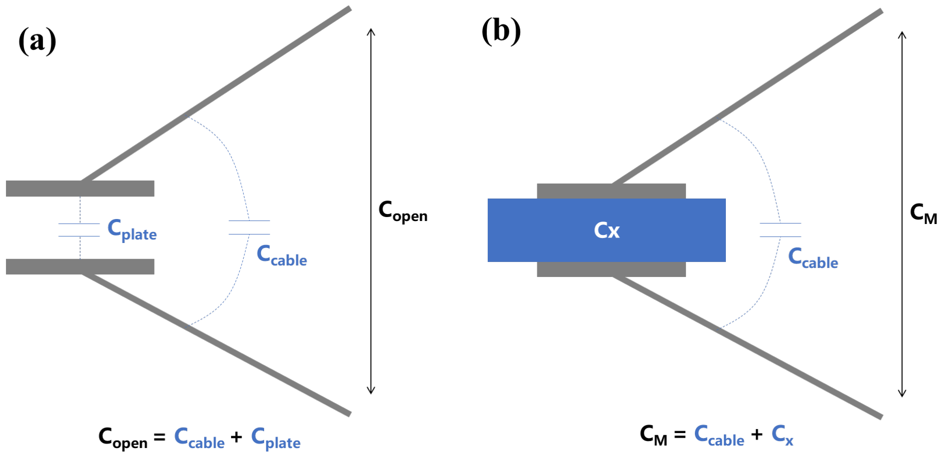

Figure S5. Diagram of the open-circuit calibration (a) and load offset calibration (b).

(c) Load offset calibration

We place the PVDF-HFP standard sample (the dielectric constant of the sample is recorded by using a commercially available equipment as $\varepsilon_s = 14.37$ at 1 kHz) on the lower plate, and the upper plate descends at a speed of 0.1 mm/s. When the value of the stress sensor reaches 0.1 N, the plate stops moving. The thickness value is d = 0.1 ± 0.002 mm, the effective area is S = 5.5 × 2.8 = 15.4 mm^2, and the capacitance measurement is C_m = 21.2 pF. The capacitor of the sample is: $C_x = \varepsilon_0 \cdot \varepsilon_s \cdot S \cdot d^{-1}$ = 19.6 pF. Therefore, C_{cable} should be $C_{cable} = C_m - C_x$ = 21.2 pF – 19.6 pF = 1.6 pF.

We also ignored the edge effect, i.e., the electric field at the edge of the electrode plates is distorted and shall introduce error in evaluating the capacitance.

According to previous reports [Eur. J Appl. Math. 32, 226-241 (2021)], the capacitance by considering edge effect could be approximated as $\frac{C}{C_{\text{ideal}}} = 1 + \frac{ln(2\pi\lambda)+1}{\pi\lambda}$, where λ is the ratio between the length and distance of the plate. In our case, the length is about 2.8 mm, and the distance is nearly 0.1 mm, which results in λ of ~ 28. Therefore, $C \approx 1.06\ C_{ideal}$. Considering that the dielectric constant of the measured TPU sample changes from ~8 to ~2.9, the correction due to the edge effect is very small.

Through calibration using the standard samples, we obtained the calibration formula for relative permittivity as:

$$\varepsilon_x = \frac{C_x d}{\varepsilon_0 S} = \frac{(C_m - C_{\text{cable}})d}{\varepsilon_0 S} \qquad (3)$$

As we measured the complex capacitances, the dielectric loss factor (tanδ) came simultaneously in the impedance meter.

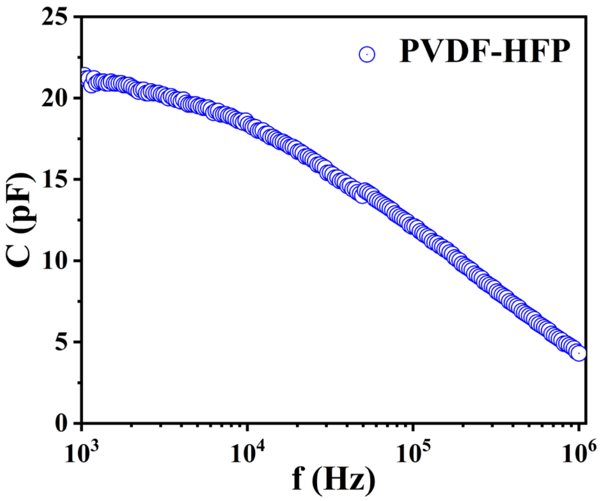

Figure S6. Fluorine elastomer standard sample capacitance test.

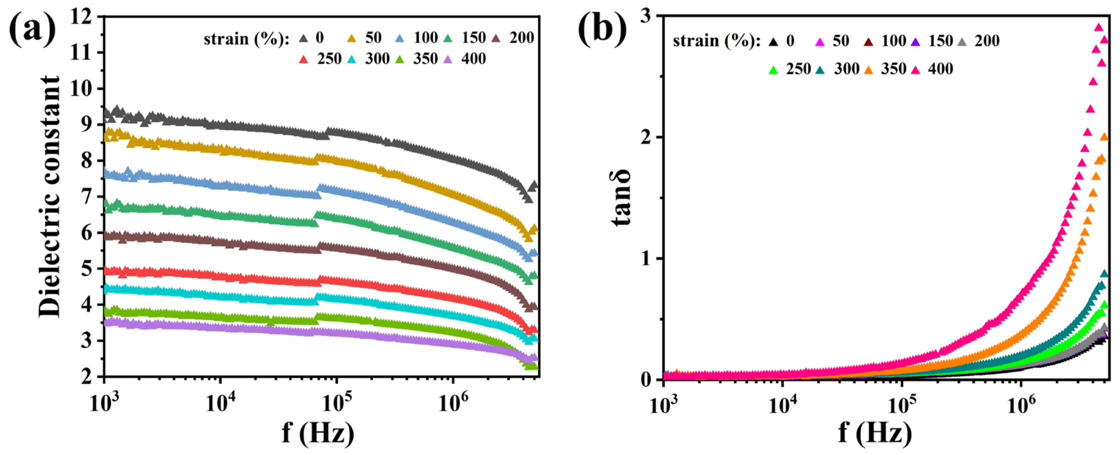

Figure S7. The frequency dependent dielectric constant (a) and loss angle tangent (b) under different strains.

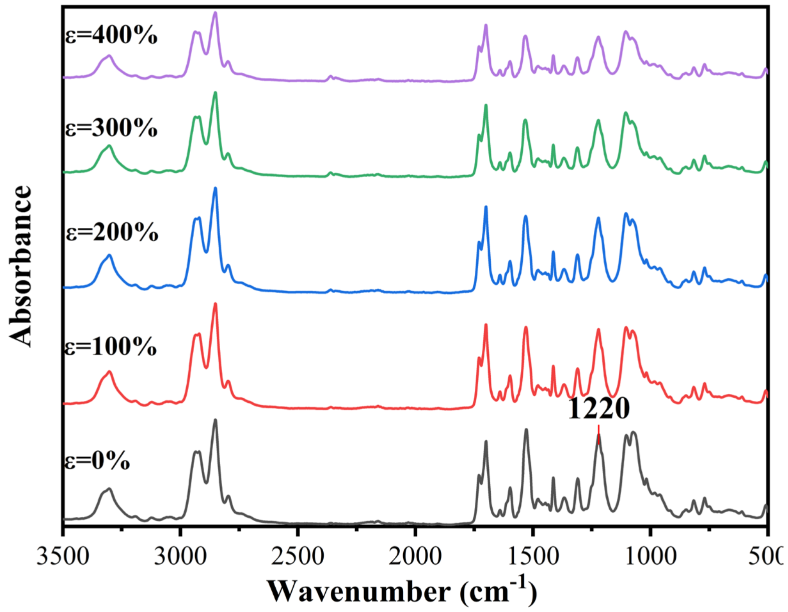

Figure S8. FTIR of TPU under different stretching strains (ε).

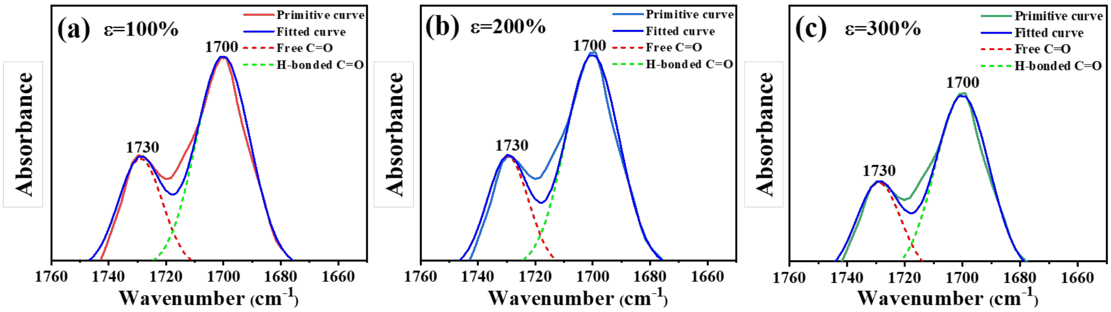

Figure S9. FTIR spectra of TPU at strain of 100% (a), 200% (b), and 300% (c), respectively.

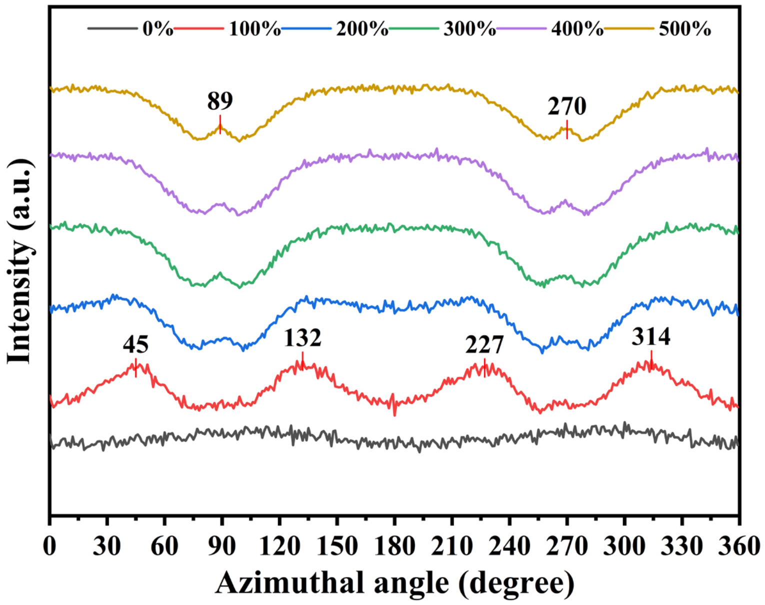

Figure S10. Azimuth angle dependence of the 1D-SAXS intensity of TPU under different strains.

Table S1. Displacement of scattering peaks (q = 1.45 $Å^{-1}$ and q = 1.55 $Å^{-1}$) under different tensile states.

Strain (%)	Peak 1 ($Å^{-1}$)	Peak 2 ($Å^{-1}$)
0	1.45 ±0.005	1.55 ±0.005
100	1.44 ±0.005	1.55 ±0.005
200	1.44 ±0.005	1.55 ±0.005
300	1.44 ±0.005	1.55 ±0.005
400	1.45 ±0.005	1.55 ±0.005
500	1.45 ±0.005	1.55 ±0.005
